# Development and Characterization of Pectin Films with *Salicornia ramosissima*: Biodegradation in Soil and Seawater

**DOI:** 10.3390/polym13162632

**Published:** 2021-08-07

**Authors:** Daniela G. M. Pereira, Jorge M. Vieira, António A. Vicente, Rui M. S. Cruz

**Affiliations:** 1 Department of Food Engineering, Institute of Engineering, Campus da Penha, Universidade do Algarve, 8005-139 Faro, Portugal; a48426@ualg.pt; 2 CEB—Centre of Biological Engineering, Campus de Gualtar, University of Minho, 4710-057 Braga, Portugal; jorgevieirabcd@gmail.com (J.M.V.); avicente@deb.uminho.pt (A.A.V.); 3 MED—Mediterranean Institute for Agriculture, Environment and Development, Faculty of Sciences and Technology, Campus de Gambelas, Universidade do Algarve, 8005-139 Faro, Portugal

**Keywords:** biobased materials, biodegradable, food packaging, pectin film, physicomechanical, *Salicornia ramosissima*, sustainability

## Abstract

Pectin films were developed by incorporating a halophyte plant *Salicornia ramosissima* (dry powder from stem parts) to modify the film’s properties. The films’ physicomechanical properties, Fourier-transform infrared spectroscopy (FTIR), and microstructure, as well as their biodegradation capacity in soil and seawater, were evaluated. The inclusion of *S. ramosissima* significantly increased the thickness (0.25 ± 0.01 mm; control 0.18 ± 0.01 mm), color parameters a* (4.96 ± 0.30; control 3.29 ± 0.16) and b* (28.62 ± 0.51; control 12.74 ± 0.75), water vapor permeability (1.62 × 10^−9^ ± 1.09 × 10^−10^ (g/m·s·Pa); control 1.24 × 10^−9^ ± 6.58 × 10^−11^ (g/m·s·Pa)), water solubility (50.50 ± 5.00%; control 11.56 ± 5.56%), and elongation at break (5.89 ± 0.29%; control 3.91 ± 0.62%). On the other hand, L* (48.84 ± 1.60), tensile strength (0.13 ± 0.02 MPa), and Young’s modulus (0.01 ± 0 MPa) presented lower values compared with the control (L* 81.20 ± 1.60; 4.19 ± 0.82 MPa; 0.93 ± 0.12 MPa), while the moisture content varied between 30% and 45%, for the film with *S. ramosissima* and the control film, respectively. The addition of *S. ramosissima* led to opaque films with relatively heterogeneous microstructures. The films showed also good biodegradation capacity—after 21 days in soil (around 90%), and after 30 days in seawater (fully fragmented). These results show that pectin films with *S. ramosissima* may have great potential to be used in the future as an eco-friendly food packaging material.

## 1. Introduction

Packaging plays a key role in containing and protecting food from external influences, such as microorganisms, oxygen, and odors, among others. However, plastic packaging has a negative environmental impact on land and sea, since it generates huge amounts of solid waste. The reduction of this waste can be achieved with the development of new biodegradable packaging systems [1]. In recent decades, a considerable number of packaging films have been developed using biopolymers, such as proteins, and polysaccharides. More recently, the combination of clays, nanostructures, and other innovative materials has been studied for novel packaging applications [2,3,4,5,6,7,8,9]. In addition to biopolymers, plant-derived bioactive compounds—such as essential oils, minerals, carotenoids, vitamins, and polyphenols, among others—have been used to modify the films’ antioxidant, antimicrobial, and physicomechanical properties [10,11,12,13,14,15].

Polysaccharides (e.g., chitosan, cellulose, starch, alginate, and pectin) have gained attention because of their good film-forming capacity [16,17]. Pectin is a heteropolysaccharide found in fruits and vegetables. It is used in food products as a stabilizing and gelling ingredient. These gelling characteristics, together with those of biocompatibility and biodegradability, make pectin an ideal biomaterial for several applications, as in the case of the pharmaceutical and food industries [18]. Several studies have reported the development of pectin films with the incorporation of different compounds. In the study reported by Makaremi et al. [18], pectin–alginate films incorporated with ascorbic and lactic acids were developed, and showed the potential to be used in food packaging. In another study, Chaiwarit et al. [19] developed a pectin film loaded with clindamycin hydrochloride to modify the film’s properties and use it as a topical drug delivery system. Nogueira et al. [20] presented a review of several methods of incorporating plant-derived bioactive compounds, among others, into pectin films for application in food packaging. The incorporation of green coffee oil and γ-aminobutyric acid residues affected the color of the films. The same review presented pectin films with *Acca sellowiana* waste byproducts (feijoa peel flour) with increased thickness and different mechanical properties.

An interesting application in the development of pectin films could be the incorporation of *Salicornia ramosissima*, a halophyte plant very rich in sodium, magnesium, potassium, calcium, and manganese. Moreover, this plant also presents good antioxidant properties [21]. Recently, this plant started to be produced by hydroponic systems, and generates a considerable number of byproducts after the cutting process. Thus, the use of this plant as a natural additive could provide a pectin film with modified properties, with a potential preservation effect mainly due to the presence of salts, and could thus be a relevant outcome to obtain an effective biodegradable active packaging material, while also contributing to the valorization of a byproduct.

To our knowledge, no study is available in the literature reporting the use of *S. ramosissima* as an additive in biodegradable packaging films. Thus, the objectives of this study were to develop and characterize a pectin film incorporating a halophyte plant—*S. ramosissima*—as well as to evaluate its biodegradation capacity in soil and seawater.

## 2. Materials and Methods

### 2.1. Materials

Apple pectin (50–70% degree of esterification; MW = 60,000–130,000 g mol^−1^) was purchased from Sigma (Algés, Portugal). Glycerol and NaBr were purchased from JMGS (Odivelas, Portugal). *Salicornia ramosissima* dried powder (from cut stem parts; particle size < 63 μm; moisture content = 7%; lipids 0.9 g, carbohydrates 4.5 g, fiber 16 g, proteins 18 g, and salt 47 g per 100 g) was kindly supplied by RiaFresh (Faro, Portugal). The soil (Eco Grow) was purchased from AKI (Faro, Portugal).

### 2.2. Preparation of the Films

Pectin films were developed based on the method reported by Mendes et al. [22], with some modifications. First, 0.5 g of apple pectin was added to 25 mL of water previously heated to 75 °C and stirred for 10 min. Next, the film-forming solution was homogenized (Ultra-Turrax T25, Janke & Kunkel, Staufen, Germany) at 20,500 rpm for 5 min, and then 1.5 mL of glycerol was added and mixed with magnetic stirring for 1 h at 40 °C.

The pectin films incorporating *S. ramosissima* were prepared by adding 0.75 g (2.7% *w*/*w*) of dried, fine *S. ramosissima* powder (based on preliminary tests to obtain a film with homogeneous appearance) to 25 mL of water previously heated to 75 °C and stirred for 10 min. After this period, the same methodology was followed as previously used for pectin films. Then, the film-forming solutions were degassed for 5 min and placed to dry in Petri dishes for 48 h at 25 °C. After drying, the films were cut and stored at 25 °C and 57% relative humidity (obtained using a saturated NaBr solution) before analysis.

### 2.3. Thickness and Water Vapor Permeability (WVP)

Three thickness measurements were randomly taken for each testing sample at different points with a digital micrometer (No. 293-5, Mitutoyo, Kawasaki, Japan). Mean values were used to calculate the WVP. The WVP of pectin films was determined gravimetrically, using the ASTM E96-92 procedure, with some modifications [23]. The permeation cell was filled with 50 mL of distilled water to generate a 100% RH (2337 Pa vapor pressure at 20 °C), and the film was sealed on the top of the cells. Then, the cells were weighed using an analytical balance (AE200, Mettler Toledo, Barcelona, Spain) and placed inside a desiccator containing silica (0% RH; 0 Pa water vapor pressure; the air circulation was kept constant by using a fan inside the desiccator). The tests were conducted in triplicate, and changes in weight of the cells were recorded at intervals of 2 h to record moisture loss over time until a steady state was reached. The WVP (g m^−1^ s^−1^ Pa^−1^) of the films tested was determined by the following equation:

WVP = (WVTR × X)/∆P(1)

where WVTR = water vapor transmission rate (g m^−2^ s^−1^) through the film, calculated from the slope of the curve divided by the film’s area; X = the film’s thickness (m); and ΔP = the partial vapor pressure difference (Pa) across the two sides of the film.

### 2.4. Color and Opacity

Color measurements were performed using a colorimeter (Minolta CR 400, Tokyo, Japan) previously calibrated with a standard white tile (EU certified; L* = 84.67, a* = −0.55, and b* = 0.68)), which recorded the spectrum of reflected light to determine the parameters L*, a*, and b*. The opacity of the samples was calculated based on the method reported by Martins et al. [24], as the relationship between the opacity of each sample in a black standard (Yb) and the opacity of each sample in a white standard (Yw), as can be seen in the equation:

Opacity (%) = Yb/Yw × 100(2)

Three measurements were taken from each sample, and three samples from each film were tested.

### 2.5. Solubility and Moisture Content

The water solubility and moisture content of the films were determined according to the method reported by Casariego et al. [25]. The film solubility in water was determined as the percentage of soluble material after 24 h of immersion in water. A disk of the film (2-cm diameter) was dried in an oven at 105 °C until constant weight to obtain the initial dry matter of the films (this part of the method allowed determining the films’ moisture content). Then, the sample was immersed into 50 mL of deionized water and gently shaken (20 °C, 24 h). At the end of the 24 h, the insolubilized films were filtered and dried in a drying oven (105 °C, 24 h) to determine the weight of the dried matter that was not solubilized in water. Three replicates were obtained for each sample. The water solubility (%) of the films was calculated as follows:

Solubility (%) = (Mi − Mf)/Mi × 100(3)

where Mi is the initial mass and Mf is the final mass of the sample.

### 2.6. Texture Measurements

Mechanical properties were evaluated in a texture analyzer (TA.TX Plus Texture Analyzer) with the software “Exponent version 6.1.16” (Stable Micro Systems, Godalming, Surrey, UK), according to the ASTM D 882 standard method [26].

A scalpel was used to cut the samples into 20-mm-wide, 100-mm-long strips, which were mounted between tensile grips. The initial grip separation and the crosshead speed were set at 80 mm and 1.0 mm/s, respectively. The tensile strength (force/initial cross-sectional area) and the elongation at break were computed directly from the strength curves vs. elongation curves by using the “Exponent” software. Young’s modulus (Equation (4)) was calculated as the slope of the initial linear portion of this curve. Six measurements were taken of each sample.

E = σ/ε(4)

where E represents Young’s modulus, σ is the tensile stress (force per unit area), and ε is the axial strain (deformation).

### 2.7. FTIR

ATR-FTIR was used to obtain information about the interactions between components in films. The FTIR spectra of the films were recorded with a PerkinElmer 16 PC (Boston, MA, USA), using attenuated total reflectance mode (ATR). Each spectrum resulted from 16 scans at 4-cm^−1^ resolution, for a spectral range of 400–4000 cm^−1^. All of the readings were performed at room temperature (20 °C).

### 2.8. Scanning Electron Microscopy (SEM)

The samples were characterized using a desktop scanning electron microscope. All results were acquired using the ProSuite software.

The samples were added to aluminum pin stubs with electrically conductive carbon adhesive tape (PELCO Tabs™). Samples were coated with 2 nm of Au (20 Angstrom) for improved conductivity. The aluminum pin stub was then placed inside a Phenom Standard Sample Holder (SH) and acquired with 10 kV.

### 2.9. Biodegradation Tests

#### 2.9.1. Seawater

The biodegradation test in seawater was based on the methodology used by Accinelli et al. [27], with some modifications. The films were cut (3 cm × 2 cm) and submerged in 300 mL of seawater (pH = 7.20). The samples were shaken at 150 rpm (Edmund Buhler-7400 Tubingen shaker) and 25 °C.

The films’ appearance was photographed during the time of the experiment. This test was carried out in triplicate for each sample.

#### 2.9.2. Soil

The biodegradation test in soil was based on the methodology used by Altaee et al. [28], with some adaptations. The films were cut (3 cm × 2 cm) and placed inside a perforated polyethylene net (5 cm × 4 cm; mesh opening 4 mm). The films were placed in soil (pH = 5.5–6.5; Humidity = 50–60%; Conductivity = 0.2–1.2 EC; Nitrogen = 80–150 mg L^−1^; Phosphorus = 80–150 mg L^−1^; Potassium = 80–150 mg L^−1^; Organic Matter = >70%) at a distance of 11 cm from the surface in a rectangular vase (71 cm × 26 cm × 25.5 cm), and with a distance of 5 cm between each film. The soil was watered with 500 mL of water every 7 days and maintained at 25 °C throughout the study. The films’ appearance was photographed and the area of biodegradation was measured during the time of the experiment. This test was carried out in triplicate for each sample.

### 2.10. Statistical Analyses

The results were expressed as the mean and standard deviation of at least three replicates. The experimental data were analyzed with IBM SPSS (Statistical Product and Service Solutions) version 26. The analysis was performed with the *t*-test to detect significant differences between the two types of film for each parameter. The significance level used was 0.05.

## 3. Results and Discussion

### 3.1. Physicomechanical Properties

Table 1 shows the physical and mechanical parameters of the control and the supplemented pectin films. The thickness of the film with *S. ramosissima* significantly increased (*p* < 0.05). This increase can be explained due to a greater solid mass in the supplemented film.

**Figure 1:**
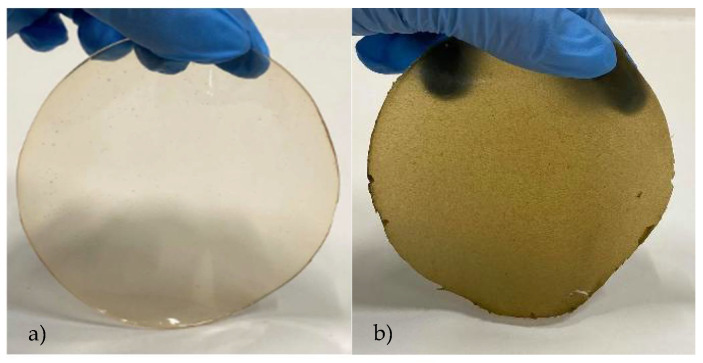
Developed films: (**a**) control film; (**b**) film with *S. ramosissima*.

According to Hosseini et al. [29], the increase in film thickness may be related to the increase in dry matter content. In terms of color, the control film was clear, colorless, and lighter, as expressed by the highest L* parameter. On the other hand, the incorporation of *S. ramosissima* led to darker and brownish-green films, presenting lower values of the L* parameter and an increase in the a* and b* parameters (Figure 1). These results, and the increase in the film’s opacity, are associated with the existing pigments in the dried powder—mainly pheophytins—obtained after drying *S. ramosissima* [30]. In another study reported by Nisar et al. [31], pectin films enriched with thinned young apple polyphenols were produced, and the authors also reported a decrease in the L* values and an increase in the a* and b* values. In another study by Sganzerla et al. [32], Brazilian pine seed starch and pectin films with the incorporation of *Acca sellowiana* extracts also showed lower L* values, together with higher a* and b* parameters.

The film with *S. ramosissima* showed a slight increase in the WVP. This increase may be related to the presence of salts and, thus, to the greater availability of hydroxyl groups to bind to water molecules [33], as well as to the film’s capacity to absorb more water molecules due to its higher mass and porous structure [34]. In a study reported by Chen et al. [35], the WVP also presented the highest values in pectin-/tara gum-based edible films with ellagitannins from the unripe fruit of *Rubus chingii* Hu. On the other hand, Nisar et al. [31] showed that the WVP decreased with the addition of components rich in polyphenols. According to the authors, the mobility of pectin chains was negatively affected by the interaction with polyphenols, so that the diffusion or penetration of water molecules through membranes was reduced, with a decrease in hydrophilic groups. The film with *S. ramosissima* also presented a higher water solubility compared with the control film. Aside from any modification to the films’ structure, the presence of salts such as sodium, magnesium, or calcium—which are soluble in water [21]—also contributed to a higher solubility of the film in water. According to Nisar et al. [31], the higher values of solubility in water and the degree of swelling of the films can be attributed to the presence of hydrophilic groups; these authors reported a behavior of their system that is identical to the one reported in our study, with a significant increase (*p* < 0.05) in solubility after the addition of apple polyphenols.

The moisture content also showed significant differences (*p* < 0.05) between the control film and the film with *S. ramosissima*. This difference is related to the higher mass of dry matter present in the film with *S. ramosissima*, contributing to lower moisture content. According to Pereda et al. [36], the moisture content is related to the total void volume occupied by water molecules in the network microstructure of the film. Similar results were obtained by Yehuala and Emire [37], where the addition of *Aloe debrana* extract and papaya leaf extract affected the moisture content of gelatin films.

Regarding the films’ mechanical properties, the control film presented a more resistant structure, showing a higher value of tensile strength, while the film with *S. ramosissima* was more brittle/fragile. On the other hand, the elongation at break was higher for the film with *S. ramosissima*. According to Shaw et al. [38], the decrease in film resistance (tensile strength) and the increase in stretching capacity (elongation at break) can be attributed to the reduction in the number of intermolecular crosslinks between pectin molecules within the films, thus contributing to a weaker material. Similar results to this study were obtained by Gouveia et al. [39], who reported that the addition of CHCl to the pectin films caused a decrease in traction resistance. The results of Meerasri and Sothornvit [40] also showed the same behavior, with a decrease in tensile strength and an increase in elongation at break with the addition of γ-aminobutyric acid and glycerol. Moreover, Kang et al. [41] reported similar results to the ones obtained in the film with *S. ramosissima*. Pectin–polyvinyl alcohol–glycerol films combined with gamma irradiation and CaCl_2_ immersion presented tensile strength values between 0.09 and 0.27 MPa, and elongation at break values between 1.02 and 3.45%.

### 3.2. Fourier-Transform Infrared Spectroscopy (FTIR)

**Figure 2:**
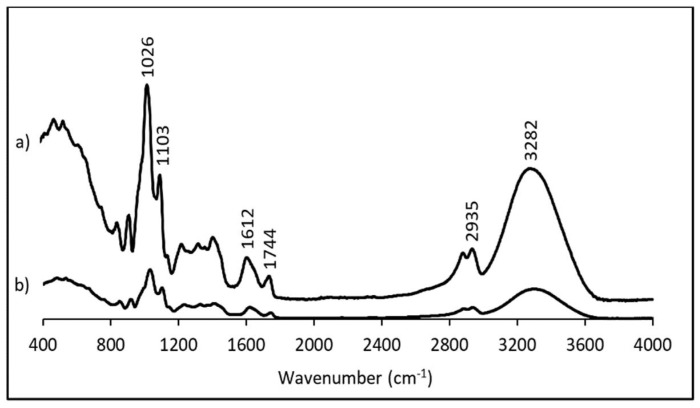
FTIR spectra: (**a**) control film; (**b**) film with *S. ramosissima*.

FTIR spectroscopy was performed to determine the intermolecular interactions within the film matrix. The FTIR spectra of the control film and the pectin film with *S. ramosissima* are shown in Figure 2. A broad peak ranging from 3700 to 3000 cm^−1^ corresponds to the stretching of O–H because of hydrogen bonding interactions in the galacturonic acid [42]. The peak at 2935 cm^−1^ is attributed to stretching of C–H bonds [43], while the bands at 1744 and 1612 cm^−1^ are attributed to the absorptions by esterified and free carboxyl groups of pectin, respectively [44,45]. Moreover, the bands at 1103 and 1026 cm^−1^ were assigned to C–O–C stretching vibrations of the polymer chain structure [46]. In general, the pectin film with *S. ramosissima* exhibited a similar pattern compared to the control film, but showed a weaker response. *S. ramosissima* dry powder is a complex mixture of diverse salts and cellulosic compounds, and its incorporation probably contributed to weakening the intermolecular forces between the chains of adjacent macromolecules [47]. This result is corroborated, as previously referred, by the lower tensile strength obtained for the film with *S. ramosissima*.

### 3.3. Scanning Electron Microscopy (SEM)

**Figure 3:**
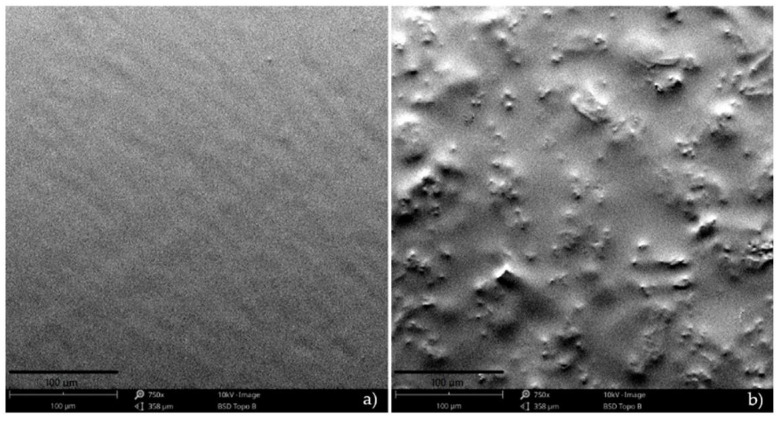
SEM micrographs: (**a**) control film; (**b**) film with *S. ramosissima* (scale bar = 100 μm).

SEM micrographs of the films’ surface are presented in Figure 3. The pectin film presented a homogeneous, compact, and smooth surface, while the film with *S. ramosissima* presented a heterogeneous and rough surface. This result is related to the presence of salts (mainly sodium, magnesium, and potassium) from *S. ramosissima* powder that changed the structure of the pectin film. The possible presence of faults or microholes also contributed to the lower tensile strength values obtained, and facilitated the migration of water vapor, causing an increase in the WVP [48], as previously shown in Table 1.

### 3.4. Biodegradation Properties

#### 3.4.1. Seawater

**Figure 4:**
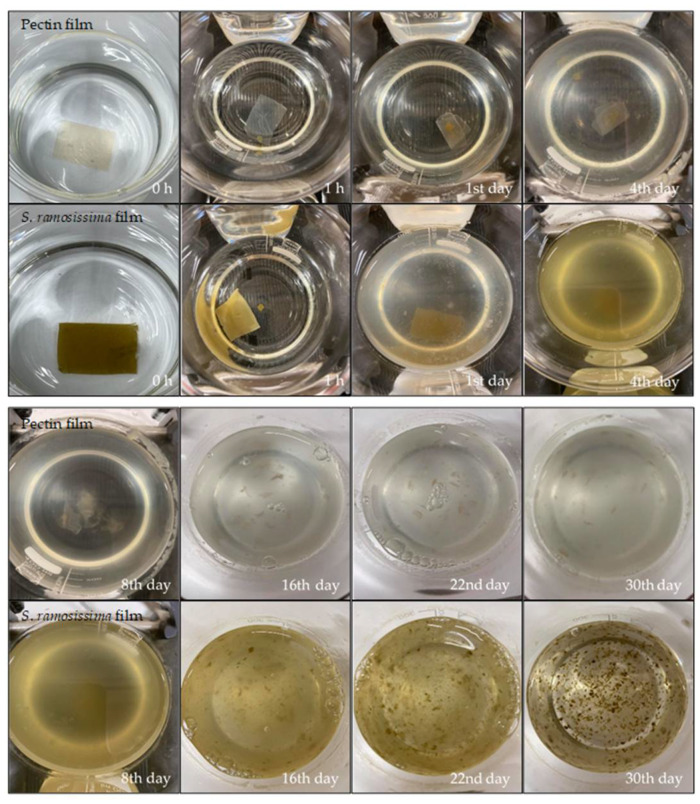
Biodegradation test in seawater.

During the biodegradation test in seawater, both films underwent several changes (Figure 4). After the first day, the samples kept their initial appearance, and after this period the seawater in which they were submerged began to show signs of clouding for the film with *S. ramosissima*, while for the control film this change was not so evident. This result can be explained by the transfer of salts and pigments from the film with *S. ramosissima* to the seawater.

On the 8th day, it was possible to verify that both films began to fragment into small pieces. In this period, the clouding of the water for the control film was also noticeable, possibly caused by the release of pectin. On the 16th day, greater changes were observed in the films’ structure, as they began to dissolve considerably in the seawater. This can be explained by the swelling of the film, as both the swelling and the solubility of the film can directly affect the water resistance properties of the films, particularly if it occurs in a humid environment [31].

On the 22nd day, the seawater began to lose signs of clouding, especially for the film with *S. ramosissima*. After 30 days, it was found that both films were quite fragmented, presenting a “flaky” appearance, and completely losing their initial rectangular shape and, consequently, most of their initial structure. The rate of biodegradation of the film is related to different factors, including the swelling, the movement/agitation of the seawater, the presence of microorganisms in the seawater, the ratio volume of seawater/film, and the existence of oxygenation [27,49]. Data obtained by Alvarez-Zeferino et al. [49] corroborate those of this study by verifying low levels of biodegradation in the first days of testing and presenting a high rate of loss of physical integrity. Furthermore, Nakayama et al. [50] performed experiments to test the biodegradation of aliphatic polyesters in seawater; the authors concluded that, aside from microorganisms, several factors—such as physicochemical effects from the sunlight, waves, and inorganic salts, among others—contribute to faster biodegradation in seawater.

#### 3.4.2. Soil

**Figure 5:**
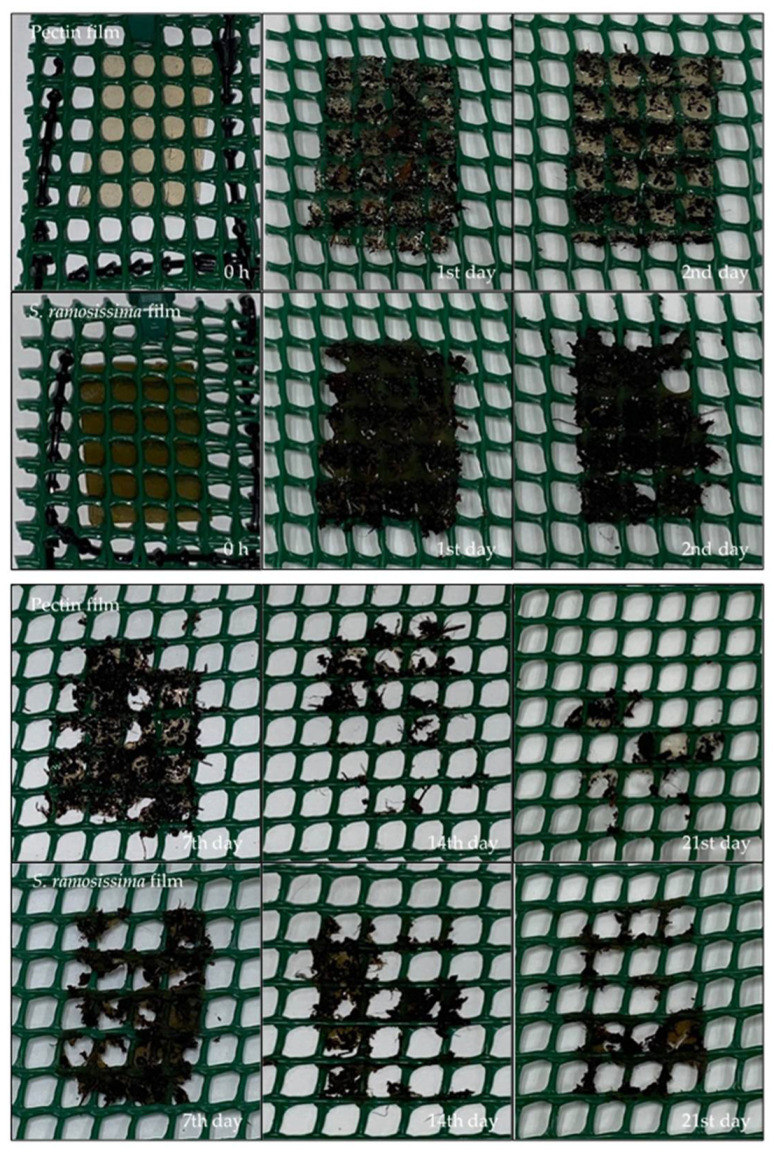
Biodegradation test in soil.

After 24 h in soil, there were no changes in the films’ structure, although the films showed signs of having some water absorption (Figure 5). After two days, the control film remained identical compared with day 0; however, the film with *S. ramosissima* absorbed water from the soil, and began to show some changes in its structure. This result is related to the fact that the film with *S. ramosissima*, as previously noted, presented a higher solubility in water.

After 14 days, there was a loss of more than 50% of the structure in both films, and after 21 days this loss was ~90%—the value indicated by the European Standard EN 13432 [51] for packaging to be considered biodegradable by biological action in a period of 6 months. This degradation is related, on the one hand, to the action of microorganisms existing in the soil. According to Shah et al. [52], the microorganisms responsible for biodegradation are bacteria and fungi; *Acidovorax facilis*, *Aspergillus fumigatus*, *Comamonas* sp., *Pseudomonas lemoignei*, and *Variovorax paradoxus* are among those normally found in soil. Our results are in agreement with the study reported by Jaramillo et al. [53], in which cassava starch films presented signs of biodegradation after 6 days. After 12 days, there was a greater change in the decomposition of films. On the other hand, the particular characteristics of the soil—such as the availability of phosphorus, which contributes to a higher load of fungi—are also responsible for the biodegradation phenomenon [54].

The degradation mechanism can first result from a variety of physical and biological forces—such as heating/cooling, freezing/thawing, or wetting/drying—causing mechanical damage, such as the cracking of the polymeric materials. In our study, the presence of water contributed to the initial breakdown of the films. Then, during degradation, depolymerization occurs when the extracellular enzymes from the microorganisms break down the polymer, yielding short chains or smaller molecules—e.g., oligomers, dimers, and monomers—that are small enough to pass the semipermeable outer bacterial membranes. These short-chain molecules are then converted to biodegradation end-products, such as CO_2_, H_2_O, and biomass [52,55].

In general, biodegradation in both soil and seawater was practically obtained in a short period, with an average degradation rate of 4.3% and 3.3% per day in soil and seawater, respectively. Conversely, plastic or even paper packaging degradation is a very slow process, and it can take several years for those materials to be fully degraded, depending on the type of plastic or paper and the used conditions [56]. According to Chamas et al. [57], low-density polyethylene (LDPE) bags are estimated to decompose by 50% after 4.6 and 3.4 years, in inland (buried) and marine environments, respectively. In a study reported by Olaosebikan et al. [58], by the 10th–12th week of exposure in soil, brown newspapers started to degrade, and only pieces of the papers were found remaining, while plastic bags had thinned off and become transparent.

## 4. Conclusions

The inclusion of *S. ramosissima* led to the development of opaque films with improved elongation at break (more flexible), but with less tensile strength (less rigid)—the latter being one of the films’ limitations. Although the films presented a homogeneous appearance, the SEM analysis revealed a heterogeneous and rough surface across the film matrix. These modifications, as verified by FTIR analysis, could be credited to the interaction between functional groups of pectin and *S. ramosissima*. Generally, the properties are promising, and the presence of salts in the films may also contribute to a preservation effect, although further characterization and improvements of the antimicrobial and antioxidant properties are necessary to achieve the desired features. Finally, the biodegradation results demonstrated that pectin films with *S. ramosissima* can also be utilized as an eco-friendly food packaging material.

## Figures and Tables

**Table 1:** Physico-mechanical parameters of the developed films.

	Control Film	Film with *S. ramosissima*
Thickness (mm)	0.18 ± 0.01 ^a^	0.25 ± 0.01 ^b^
Color		
L*	81.20 ± 0.70 ^a^	48.84 ± 1.60 ^b^
a*	3.29 ± 0.16 ^a^	4.96 ± 0.30 ^b^
b*	12.74 ± 0.75 ^a^	28.62 ± 0.51 ^b^
Opacity (%)	10.87 ± 0.05 ^a^	30.04 ± 1.49 ^b^
Water vapor permeability (g/(m·s·Pa))	1.24 × 10^−9^ ± 6.58 × 10^−11 a^	1.62 × 10^−9^ ± 1.09 × 10^−10 b^
Water solubility (%)	11.56 ± 5.56 ^a^	50.50 ± 5.00 ^b^
Moisture content (%)	45.79 ± 0.76 ^a^	30.11 ± 4.41 ^b^
Elongation at break (%)	3.91 ± 0.62 ^a^	5.89 ± 0.29 ^b^
Young’s modulus (MPa)	0.93 ± 0.12 ^a^	0.01 ± 0 ^b^
Tensile strength (MPa)	4.19 ± 0.82 ^a^	0.13 ± 0.02 ^b^

^a, b^ Different superscript letters indicate significant differences (*p* < 0.05).

## Data Availability

The data presented in this study are available on request from the corresponding author.
